# Boiling Time to Estimated Stunning and Death of Decapod Crustaceans of Different Sizes and Shapes

**DOI:** 10.3390/ani14223277

**Published:** 2024-11-14

**Authors:** Henrik Lauridsen, Aage Kristian Olsen Alstrup

**Affiliations:** 1 Department of Clinical Medicine, Aarhus University, 8200 Aarhus N, Denmark; aagealst@rm.dk; 2 Department of Nuclear Medicine & PET, Aarhus University Hospital, 8200 Aarhus N, Denmark

**Keywords:** Decapoda, crustacea, surface area, sphericity, humane killing, heating, boiling, euthanasia, allometry, micro-CT

## Abstract

The best practice for killing decapod crustaceans lacking a centralized ganglion has been debated for a century. Currently, there is a movement away from live boiling towards electrocution and mechanical splitting or spiking, which are efficient in the large commercial setting but may be unavailable and impractical for small decapods such as shrimp and prawn in the small-scale setting of, e.g., the household. Here, using carcasses of varying sizes of prawn, crayfish, lobster and green and brown crab, we used micro-CT imaging to measure surface area and sphericity in relation to body mass. Then, we measured heating profiles at the anterior ganglion and in the core of carcasses of the same species when exposed to standardized boiling regimes. We found a relationship with positive allometry between surface area and body mass for all species and a decrease in sphericity with mass. Heating times until proposed stunning (26 °C) and killing (44 °C) varied with body size and starting temperature and exceeded minutes for larger species. For a small species like prawn, times to stunning and killing by boiling are comparable to electrocution times and within the acceptable range compared to recreational killing of other sentient beings such as game mammals.

## Simple Summary

Decapod crustaceans constitute an important resource of animal protein and are harvested both in large-scale commercial fisheries as well as in small-scale recreational settings. The best practice for killing decapods—lacking a single centralized ganglion—has long been debated, and recent years has seen a tendency to abandon traditional live boiling based on animal welfare considerations. However, omnibus legislation disregarding the large span in body sizes and shapes of decapods may result in overly restrictive laws on cooking small decapods in small-scale settings, e.g., in the household, where electrocution devices are not available. Here, we first used imaging to measure the relationship between surface area and body roundness to body mass, which are important parameters for heat dissipation. Then, we measured heating profiles during boiling for carcasses of five species of decapods, representing a variation in body size and shape. We found that the time to stunning and killing was in the minutes range for larger species, but, for a small species like prawn, these events happened within 9–24 s, which was in the range of electrocution devices as well as within the time to incapacitation during other types of recreational killing of sentient animals such as game mammals.

## 1. Introduction

The first signs of hominin consumption of cooked decapod crustaceans date back to the Middle Paleolithic [1]. Today, a large number of decapod crustaceans are consumed by humans; by some estimates, yearly landings approach 5 × 10^6^ tons (~5% of global total fisheries production) and as many as 1.6 × 10^12^ individuals [2,3,4]. The vast majority of decapods consumed by humans are slaughtered in large-scale industrial facilities, but small-scale exploitation and recreational harvesting remain important routes of generating side income or as leisure-time activities in many countries and cultures [3]. Like most other sources of animal protein, the consumption of decapods involves the process of killing the animal. In most societies, it is generally required that killing should be conducted in the most humane way, i.e., causing the least amount of pain, suffering and distress to the animal and for as short a time as possible. As an example, although only applying to vertebrates (and in fact excluding amphibians and reptiles), European Union Council Regulation (EC) No 1099/2009EU, Article 3, dictates that “Animals shall be spared any avoidable pain, distress or suffering during their killing and related operations” [5]. However, in the case of decapods without a single centralized ganglion, the most humane way to terminate the animal is not obviously straightforward and has been the subject of debate for a century, especially for larger decapod crustaceans like lobster and crab [6,7,8,9,10,11].

The order Decapoda contains species ranging in size by four orders of magnitudes from, e.g., shrimps weighing a few grams to large crabs and lobsters with body masses surpassing 10 kg [12]. The diversity in the size and shape of decapods and the lack of a single centralized ganglion result in a difficult discussion on how legislation and recommendations for the killing of decapod crustaceans as a group should be constructed to provide both ethical as well as practical rules and guidelines across species that are functional in both the large-scale industrial setting and, which will be the focus here, in the small-scale setting of household cooking.

While direct cooking in boiling water remains the most traditional way of both dispatching and cooking decapods, this procedure has long been ethically problematized and in some countries made illegal since both the laws of thermodynamics as well as direct observations on decapods, especially larger species like lobster and crab, placed in boiling water show that death is not instantaneous [8,13]. Another suggested way of killing is by making the decapod unresponsive by placing it in ice-cold water or freezing before cooking; however, this may only make the process appear more humane and in fact still cause suffering [10,14,15]. Mechanical killing by splitting the animal (lobster) or spiking the main ganglia (crab) are currently recommended methods for killing decapods in some countries and by some animal welfare institutions [11,16,17]; however, in cases of smaller species such as small shrimp and prawns where hundreds or thousands of animals need to be killed to make a meal, these methods are inefficient and are unlikely to be used by most private consumers. In recent years, electric devices have been developed using electrocution to stun and kill decapods [11,14,18,19,20,21,22]. While such products can be targeted at both large-scale seafood producers as well as smaller business in the hospitality sector, e.g., seafood restaurants and shellfish mongers with a moral and/or economic incentive to document ethical handling and killing of their products, they probably remain too expensive (>3000 EUR) for layman and recreational use, and, like mechanical killing, they remain impractical for killing many small decapods at the same time, such as shrimp and prawn.

Legislation on the live boiling of decapod crustaceans varies by country. In Table 1, we collate information from references and direct contact with veterinary professionals and ministerial officials (see acknowledgements) about the legal status of live boiling decapod crustaceans and any specific mentioning of large or small species in the law or comments to the law in different countries [10]. In 2018, the Swiss Confederation passed legislation on the subject of killing decapod crustaceans with indirect considerations for small species (Table 1) [23]. Here, it was specified that killing any member of the suborder Pleocyemata (i.e., excluding the other decapod suborder of Dendrobranchiata, prawns) with the exception of the infraorders of Stenopodidea (boxer shrimps) and Caridea (true shrimps) should only take place using anesthetics or if the killing method used is without pain or suffering or puts the animal into a state of insensibility and unconsciousness. With this detailed legislation, direct boiling of the generally smaller members of the Dendrobranchiata, Stenopodidea and Caridea taxons of decapod crustaceans remains legal. Similar protection of decapod crustaceans from live boiling exists in Norway, New Zealand and the United Kingdom (Table 1) [10], but, to our knowledge, without consideration for differences between species of different shapes and sizes. In regions of Italy and Belgium as well as in Denmark, decapod crustaceans also receive some legislative protection from live boiling, e.g., in comments to the law (Table 1). But, excluding the more detailed Swiss legislation and the specific mentioning of lobsters in Belgian, Italian and Danish comments/opinions, decapod crustaceans are in general treated as one, and small species such as shrimps and prawns should in principle be treated similarly to large species such as lobsters and large crabs, although, to our knowledge, this assumption has never been tested in court. Superficially, this may seem sympathetic, but, at the small-scale level, e.g., in portions of the hospitality sector and, in particular, in the private household, it seems unlikely that the common practice of directly boiling small decapods such as shrimp and prawn will change since currently there are no other practical methods. Thus, in countries with undetailed regulations on live decapod boiling as well as other countries with plans for adopting similar undetailed legislation on this matter where decapod crustaceans are all lumped together, there is a potential problem with the principle of legality in that there is a mismatch between law and how small-scale and recreational harvesters and consumers of small decapods dispatch these in a sensible way.

Apart from a few studies on boiling time in medium-sized and large decapod crustaceans, crayfish, lobster and edible crab [14,24,25], most evidence on killing time by boiling is anecdotal. To provide a solid base for future legislators to decide sensible rules and guidelines for dispatching decapod crustaceans of different sizes and types, we set out to establish geometric relationships between body size and shape and measure the time it takes for carcasses of decapods of different body sizes and shapes to reach stunning and killing temperatures at the anterior ganglion (the cerebral ganglion, supraesophageal ganglion—also known as “brain”) and in the core of the cephalothorax. We set the stunning temperature at 26 °C, since this has evidence for lobster and brown crab [7,14], and we set a conservative killing temperatures of 44 °C based on measurements in crayfish [24], although death may occur even at a lower temperature for some species. For this study, we used species typically harvested on a large commercial scale but more importantly also on a small recreational scale in Northern Europe. We used micro-CT imaging to measure body surface area and shape (sphericity) in relation to body mass as these parameters are important for the understanding of heating times of animals of different sizes and shapes. Then, we measured heating times by placing thin needle thermometers inside carcasses and exposing these to a realistic boiling regime. When possible, we used animals of different size groups (small, medium and large) within each species, and we measured heating times in a standardized heating regime when starting at a body temperature of 0 °C, i.e., reflecting boiling after chilling in iced water; 10 °C, reflecting common sea or lake water temperature at the time of harvest in Northern Europe; and 20 °C, reflecting the storage of animals at standard room temperature.

## 2. Materials and Methods

**Figure 1:**
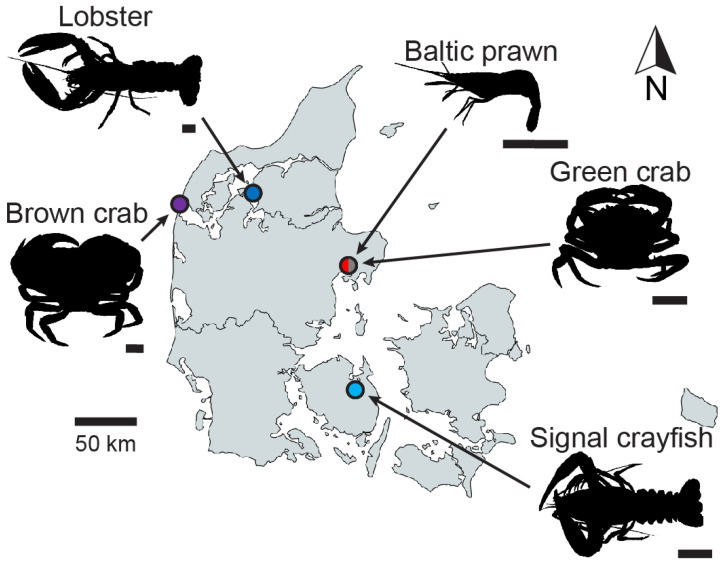
Map of the country of Denmark with collection sites for the five included species of decapod crustaceans. Scale bars under each of the representative silhouettes are 1 cm for easy size comparisons. The map was created with mapchart.net.

The decapod crustaceans included in this study were as follows: Baltic prawn *Palaemon adspersus* Rathke, 1837 (n_total_ = 54), signal crayfish *Pacifastacus leniusculus* (Dana, 1852) (n_total_ = 33), European lobster *Homarus gammarus* (Linnaeus, 1758) (n_total_ = 3) (in the following referred to as “lobster”), European green crab *Carcinus maenas* (Linnaeus, 1758) (n_total_ = 40) (in the following referred to as “green crab”) and brown crab *Cancer pagurus* Linnaeus, 1758 (n_total_ = 20). All specimens were legally fished in Danish waters (fishing license# 1060091202) in 2023 outside any closed season (the European lobster closed season in Limfjorden is 1st of July until 31st of August; the other species do not have any closed season) and strictly adhering to size regulations (European lobster: carapace length ≥ 8.7 cm). Baltic prawns and green crabs were fished using a shrimp push net and breath-hold diving, respectively, in the bay of Kalø Vig (56°16′48.6″ N 10°29′09.9″ E); signal crayfish were fished using dip nets in Davinde Sø (55°19′55.8″ N 10°32′22.7″ E); lobsters and brown crabs were collected during breath-hold diving at Ertebølle Strand (56°48′28.4″ N 9°09′38.9″ E) and Agger Tange (56°43′18.1″ N 8°12′39.9″ E), respectively (Figure 1). All applied fishing methods are gentle, and all collected specimens were in good health without any visible injuries. No crustacean species are included in the list of animals for which an animal experimental permit is required by the Danish Animal Experiments Inspectorate or Directive 2010/63/EU; and no live animal experiments were performed in this study, since all the animals were sacrificed before imaging and boiling experiments. The decision to perform the study on euthanized rather than live animals was based on the ethical consideration that neither imaging nor heating requires live animals to derive precise measurements.

The included species were used for two analyses: to establish the correlation between size and shape (body size versus surface area and sphericity) and for a heating experiment. To investigate the relationship between body mass and shape, specimens of different sizes (Baltic prawn: n = 27, signal crayfish: n = 13, green crab: n = 19, brown crab: n = 20, lobster: n = 3) were imaged using X-ray computed tomography (Table S1). Small species (Baltic prawn, signal crayfish, green crab and small specimens of brown crab) were imaged using a Medical XtremeCT system (Scanco, Brüttisellen, Switzerland) with the following parameters: X-ray tube voltage = 59.4 kVp, X-ray tube current = 119 µA, integration time = 132 ms, field-of-view = 70 × 70 × 150 mm^3^, spatial resolution = 0.082 mm isotropic, acquisition time = 1.5 h pr. scan. Large species (lobster and large specimens of brown crab) were imaged using a Canon Aquilion Prime SP system with the following parameters: X-ray tube voltage = 120 kVp, X-ray tube current = 114 mA, integration time = 1000 ms, field-of-view = 307 × 307 × 1418 mm^3^, spatial resolution = 0.6 mm isotropic, convolution kernel = FC18, acquisition time = 60 s pr. scan. Surface area was measured by image segmentation and using the Surface Area Volume module in the visual imaging software Amira version 5.3.3. An additional Baltic prawn and a green crab were used for diffusible iodine-based contrast-enhanced micro-CT imaging to visualize the main ganglia. This was performed by staining the formaldehyde fixed specimens using 2.5% Lugol’s solution (8.33 g/L I_2_ and 16.66 g/L KI in water) to visualize soft tissues and then conducting micro-CT imaging using a CoreTOM system (TESCAN GROUP, Brno, Czech Republic) equipped with a integrating detector and using the following parameters: X-ray tube voltage = 120 kVp, X-ray tube power = 15 W, integration time = 1800 ms, field-of-view = 35.4 × 49.8 × 32.2 mm^3^, spatial resolution = 0.015 mm isotropic, acquisition time = 17 h per sample.

For the heating experiment, specimens of similar size groups within species were selected to minimize variability (Table 2). Signal crayfish and green crab were divided into three size groups: “small” (considerable smaller than normal eating size), “medium” (normal eating size) and “large” (considerable larger than normal eating size). The Baltic prawns were relatively uniform in size with very few extraordinary large individuals but some small individuals; thus, only “small” and “medium” were used as size groups for this species. Brown crabs were collected in a range of sizes, in particular within the normal eating size, 500–1000 g, set as the “medium” size, but also in smaller sizes comparable to the generally much smaller green crabs; thus, a “small” group of brown crabs was created containing specimens of similar size as “large” green crabs. No brown crabs attaining very large sizes (>2 kg), which is possible for the species, were collected; thus, no “large” group was created for this species. The minimum size limitation for lobster ruled out the possibility of creating a “small” group for this species, and no really large lobsters (>3 kg) were collected; thus, only a “medium” size group was created for this species. The combination of five species, up to three size groups and four starting temperatures yielded at total of 19 separate heating groups. Since variability in heating profiles was generally low, a group size ≥4 was settled upon, except for the largest species/size groups (medium brown crab, n = 2, medium lobster, n = 3) as long heating times have already been established for these large species [8,13,14]. Body mass (all species), total length (tail tip to rostrum tip) (Baltic prawn, signal crayfish, lobster), carapace length (eye socket to posterior carapace) (Baltic prawn, signal crayfish, lobster), carapace width (green crab, brown crab) for different size groups ± standard deviation are reported in Table 2 and Table S2. Values are reported as average ± standard deviation. The study purpose of estimating time to stunning and death in differently sized and shaped decapod crustaceans to quantitatively establish a link between size, shape and heating time did not include specifically testing differences in heating times between species as this would follow well-established laws of thermodynamics; thus, no statistical testing was performed to test for differences between groups and species.

All crustaceans were euthanized by immersion in an overdose of 2 mL/L clove oil (H’ana, active ingredient: eugenol) initially dissolved in 10 mL 100% ethanol and then in the animals own medium for a minimum of two hours for small species (prawns, crayfish and green crabs) and five hours for larger species (lobster and brown crab) [26,27]. This was performed prior to thermometer insertion and boiling or imaging. For boiling experiments, the temperature of the euthanized crustaceans was adjusted to either 0 °C by placing in ice slurry, 10 °C by adequate refrigeration or 20 °C (room temperature). Fine-tipped thermometers (CDN Thin Tip Thermometer DTQ450X, Portland, OR, USA) were inserted at the anterior ganglion and/or at the body core (simultaneous recordings at both anterior ganglion and body core were possible in larger species: signal crayfish, green crab, brown crab and lobster). The sides of the thermometer tip were secured to the exoskeleton of the crustaceans and insulated from water infiltrating into the puncture wound using a small piece of gum and dropwise addition of super glue. When the crustacean cadavers reached the desired starting temperature in the core (0, 10 or 20°), they were boiled in heavily salted water (30 g/kg NaCl) according to general practice [28]. To adjust for the fact that smaller crustaceans are rarely cooked alone and the batch size will affect the initial cooling and the reheating of boiling water, a standardized boiling regime was developed and used. For each boiling, 7 kg of saltwater was brought to the point of boiling (100.5 °C). Then, a portion of saltwater was prepared at the same temperature as the crustacean cadavers with a mass of 777 g minus the mass of cadavers. In this way, the dilution factor of cold cadavers and water to boiling water was 1:9 (dilution to 10%), in our experience equaling a common cooking practice of cooking ~1 kg crustaceans in a partially filled 10 l pot. Temperature-adjusted water and crustacean cadavers were added simultaneously to the boiling water thereby lowering the water temperature to 90.5–92.5 °C depending on the initial temperature of the adjusted water. Water temperature and animal temperature was continuously recorded for one (lobsters and large brown crabs), two (green crabs and small brown crabs) and four (Baltic prawns and signal crayfish) individuals per cooking event as the water was reheated using an electric stove with an effect of 1.34 kW (3–5 min until re-boiling, depending on the initial temperature of the adjusted water) until internal animal temperature reached 99 °C. Following the standardized heating regimes, we also tested the fastest possible heating rate in six Baltic prawns by setting the start temperature close to a realistic in-house summer temperature of ~24 °C and cooking without the addition of temperature-adjusted water.

## 3. Results

### 3.1. Morphology

**Figure 2:**
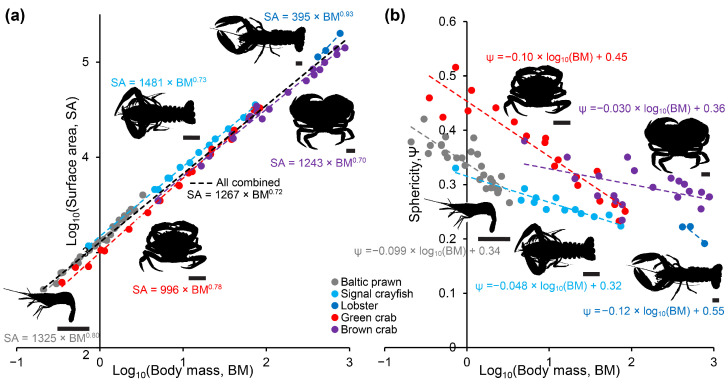
Surface area (SA) in panel (**a**) and sphericity (Ψ) in panel (**b**) over body mass (BM) in Baltic prawn, signal crayfish, European lobster, European green crab and brown crab. Power functions (**a**) and logarithmic functions (**b**) of best fits are displayed for each plot. Scale bars under each of the representative silhouettes are 1 cm for easy size comparisons.

The scaling of surface area to body mass was found to show positive allometry (scaling exponent > 2/3) for all five included species of decapod crustaceans (Figure 2a, Table S1). Generally, sphericity was closer to 1 (i.e., perfectly spherical) for the two species of crabs than the three more elongated species of prawn, crayfish and lobster, and sphericity generally decreased with body size (Figure 2b, Table S1). Due to the low number of lobsters included, results for this species should be viewed with caution.

**Figure 3:**
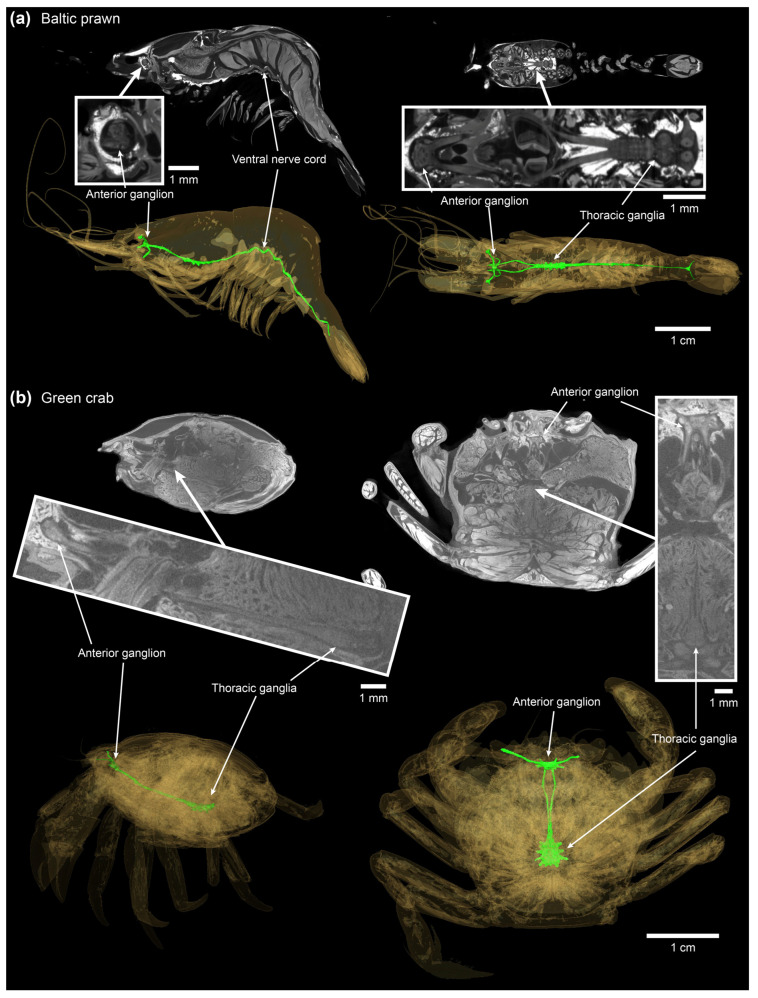
Central ganglia in Baltic prawn and European green crab revealed by diffusible iodine-based contrast-enhanced micro-CT imaging. Top parts of each panel display sagittal (**left**) and coronal (**right**) virtual sections through the specimens. Bottom parts display a volume rendering of the specimen (yellow) overlaid by the central ganglia (green).

The position of anterior and central ganglia was visualized in Baltic prawn and green crab using micro-CT imaging (Figure 3). The anterior supraesophageal ganglion takes a quite superficial position in both species. In the prawn, the thoracic and abdominal ganglia and the connecting ventral nerve cord also take a superficial position along the ventral body surface of the animal. In the green crab, although still ventrally located, the posterior ganglia are located deeper and closer to the body core (Figure 3).

### 3.2. Heating Times

**Figure 4:**
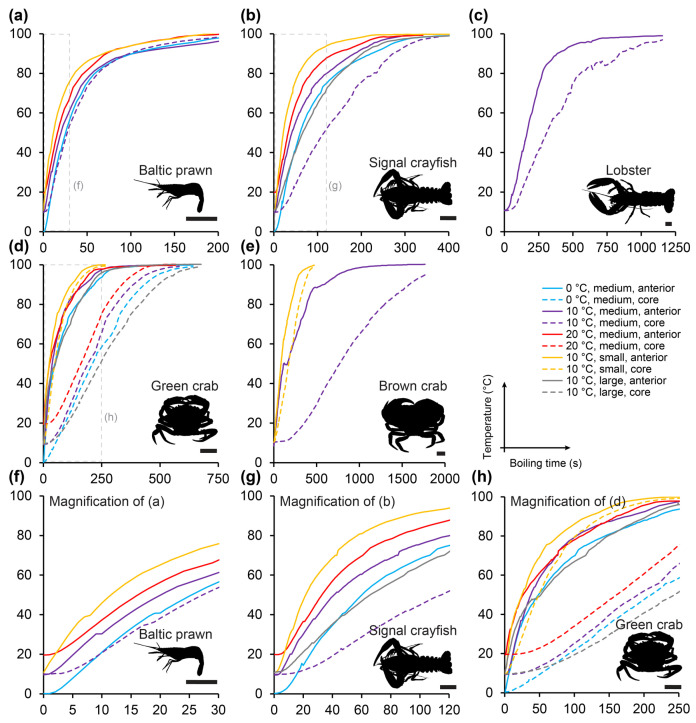
Representative heating curves (temperature over boiling time) at the anterior ganglion (solid lines) and core (dashed lines) for Baltic prawn (**a**), signal crayfish (**b**), European lobster (**c**), European green crab (**d**) and brown crab (**e**) of different sizes and starting temperatures (see color coding in the middle right panel). Panels (**f**,**g**,**h**) are magnifications of portions of panels (**a**,**b**,**d**), respectively, to better reveal temperature development early in the process of boiling.

Heating profiles varied between body shapes of the five included species of decapods (representative profiles in Figure 4, full data in Table S2). Both the anterior ganglion temperature and the core temperature generally followed a sigmoid pattern, with an initial lag phase followed by a phase of rapid increase in temperature and terminated by a slow transition towards the boiling point (Figure 4). However, for smaller species and size groups (Baltic prawns and small signal crayfish and green crabs) the sigmoid curve shape was left-shifted, thus showing a very rapid increase in temperature upon exposure to the heating bath (Figure 4a,b,d).

**Figure 5:**
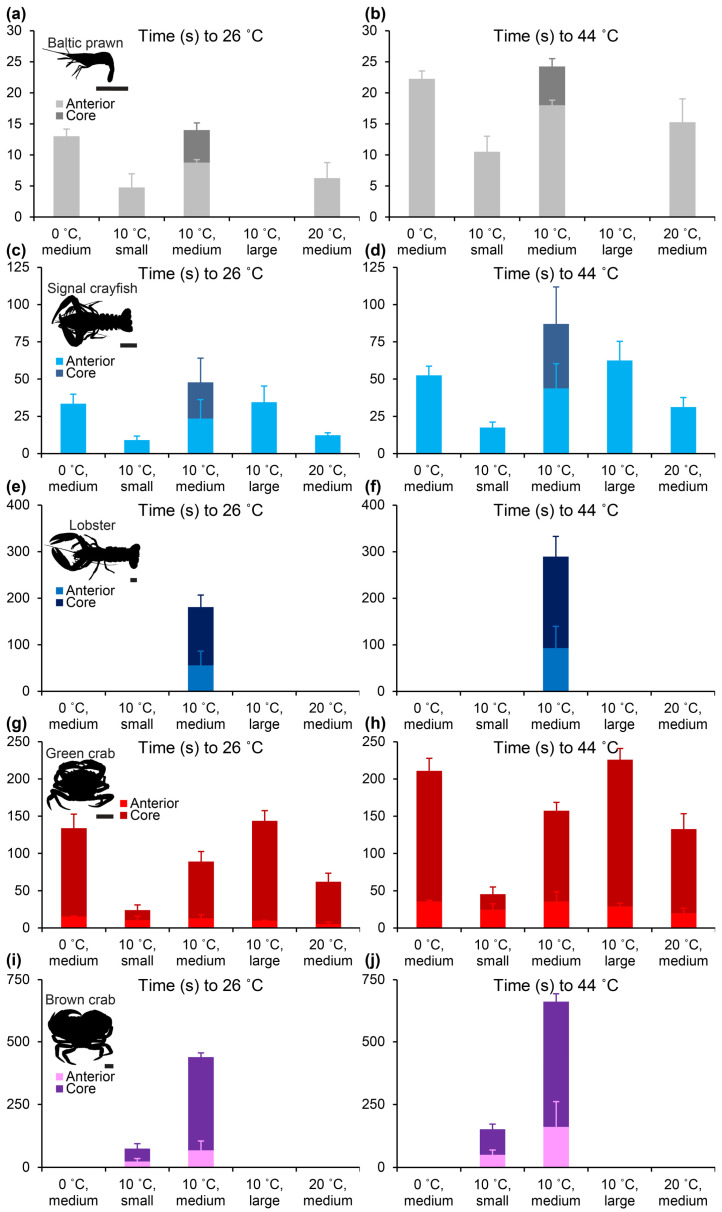
Average times ± standard deviation to reach stunning temperature, defined as 26 °C (left panels), and killing temperature, defined as 44 °C (right panels), at the anterior ganglion (light colors) and core (dark colors) in Baltic prawn (**a**,**b**), signal crayfish (**c**,**d**), European lobster (**e**,**f**), European green crab (**g**,**h**) and brown crab (**i**,**j**) of different sizes and starting temperatures. For all species and size groups n = 4, except for medium-sized lobsters where n = 3 and medium-sized brown crabs where n = 2.

Average heating times logically followed thermodynamic reasoning. Large species and size groups heated slower than smaller species and size groups, and the generally more superficially located anterior ganglion heated more rapidly than the deeper core (Figure 5, Table S2). The anterior ganglion of medium-sized Baltic prawns reached 26 °C (potential stunning temperature) after 8.8 ± 0.5 s (t_10->26 °C, ant._) when heated from an average spring sea temperature of 10 °C and a killing temperature, 44 °C, after 18.0 ± 0.8 s (t_10->44 °C, ant._). The core temperature of this group reached 26 °C after 14 ± 1.2 s (t_10->26 °C, core_) and 44 °C after 24.3 ± 1.3 s (t_10->44 °C, core_). In comparison, the much larger and more spherical medium-sized brown crabs reached 26 °C at the anterior ganglion after 68.0 ± 36.8 s (t_10->26 °C, ant._) and 44 °C after 161.0 ± 100.4 s (t_10->44 °C, ant._) when similarly heated from 10 °C and, at the core, reaching 26 °C took 438.0 ± 17.0 s (~7 min) (t_10->26 °C, core_), and 44 °C took 661.5 ± 31.8 s (~11 min) (t_10->44 °C, core_).

The fastest heating was observed in the non-standardized heating experiment on medium-sized Baltic prawns which were heated from a warm room temperature (24.4 ± 0.6 °C) by direct placement in boiling water without any addition of water to mimic batch boiling. In this case, the anterior ganglion reached a temperature of 26 °C within 2.3 ± 1.2 s (t_24->26 °C, ant._) and 44 °C within 9.3 ± 2.3 s (t_24->44 °C, ant._) (Table S2).

**Figure 6:**
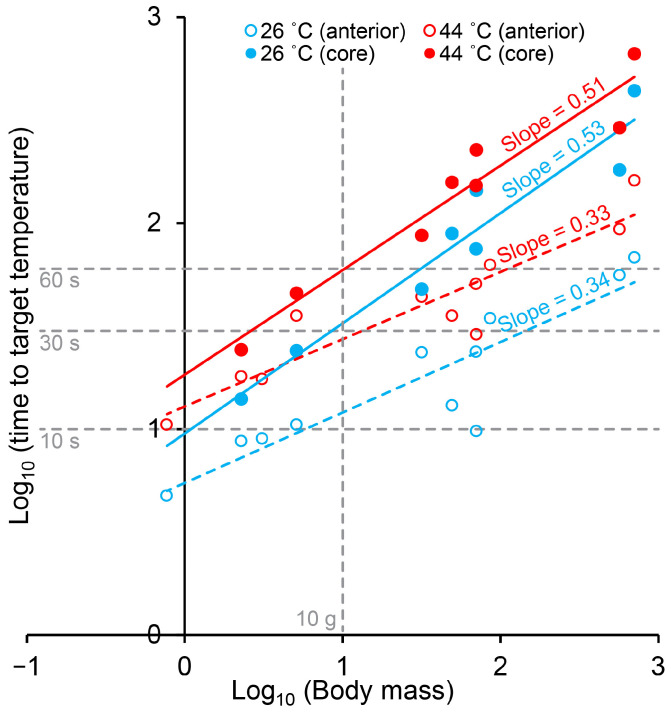
Average heating times to reach stunning (26 °C) and killing (44 °C) temperatures when heated from 10 °C plotted as a function of body mass of all included decapod species and size groups. Heating rate at the core is more dependent on body mass (larger slope) than heating rate at the anterior ganglion.

In spite of the different body shapes of the five included species of decapod crustaceans, the heating rate at both the anterior ganglion and in particular in the core followed the trend of a power function (straight line on log–log plot, Figure 6), with the heating rate being more dependent on body mass in the core (larger slope, Figure 6) than at the anterior ganglion.

## 4. Discussion

Decapoda is a highly successful and variable order of crustaceans. Although body shapes are far from similar, we found that within the caridean (Baltic prawn), astacidean (signal crayfish and lobster) and brachyuran (green and brown crab) species included in this study, body surface area generally scaled with positive allometry to body mass with scaling exponents ranging between 0.70 and 0.93 (Figure 2). The two included species of crab were, unsurprisingly, more spherical than both prawn, crayfish and lobster. The thermodynamically logical reasoning following this would predict that round decapods like crab would heat slower than more elongated shrimps and astacidean decapods, and, in spite of positive allometry (which may result from a relative enlargement of chelipeds in larger individuals), larger individuals within a species would be predicted to heat slower than smaller individuals. This logic was strongly supported by our measurements of heating times in the different species and size groups, in particular for the core measurements (Figure 4 and Figure 5 and Table S2). Larger species and size groups yielded much slower heating rates than smaller species and size groups. Animals of different species but of similar size and shape, however, demonstrated similar heating times, i.e., medium-sized Baltic prawns (2.37 ± 0.29 g) heated from 10 °C with t_10->26 °C, ant._ = 8.8 ± 0.5 s and t_10->44 °C, ant._ = 18.0 ± 0.8 s compared to small-sized signal crayfish (3.1 ± 1.8 g) heated from 10 °C with t_10->26 °C, ant._ = 9.0 ± 2.7 s and t_10->44 °C, ant._ = 17.5 ± 3.7 s and to some degree but with more variation when comparing large-sized green crabs (70.55 ± 7.14 g) heated from 10 °C with t_10->26 °C, ant._ = 9.8 ± 0.5 s, t_10->26 °C, core_ = 143.8 ± 13.6 s, t_10->44 °C, ant._ = 28.8 ± 4.5 s and t_10->44 °C, core._ = 226.0 ± 14.6 s to small-sized brown crabs (70.04 ± 8.36 g) heated from 10 °C with t_10->26 °C, ant._ = 23.8 ± 10.8 s, t_10->26 °C, core_ = 74.8 ± 19.0 s, t_10->44 °C, ant._ = 50.5 ± 18.6 s and t_10->44 °C, core._ = 152 ± 19.8 s. This can be particularly appreciated when plotting heating times to stunning and killing temperatures when starting from 10 °C for all included species and size groups (Figure 6).

Heating times to stunning and killing approached minutes in the core of large decapods, which is comparable to a previous report on brown crab [14]. Medium-sized lobsters (569.70 ± 183.66 g) heated from 10 °C showed t_10->26 °C, core_ = 3.0 ± 0.4 min and t_10->44 °C, core._ = 4.8 ± 0.7 min, and medium-sized brown crabs heated from 10 °C showed t_10->26 °C, core_ = 7.3 ± 0.3 min and t_10->44 °C, core._ = 11.0 ± 0.53 min. Although, as demonstrated by imaging the central nervous system in Baltic prawn and green crab (Figure 3), it is expected that the main portion of the central ganglia in the larger lobster and brown crab are likewise positioned more superficially than the body core. Thus, core measurements should be taken as the most conservative heating time estimate and also the most sensitive estimate in regards of body size (larger slope in Figure 6). However, it remains clear that stunning and killing by boiling is a slow process in large decapod crustaceans, which is in line with observations of escape behavior as reported in previous studies using live boiling [8,13].

The smallest of the included species, the Baltic prawn, takes an ethically interesting position. With an average size of just a little more than 2 g live weight for normal eating size and a ~28% meat yield when peeled (personal observation by author HL) hundreds to thousands of individuals are required to make an acceptable human meal. Thus, in opposition to the other four included species, the Baltic prawn represents a species where mechanical killing by splitting or spiking individual animals would certainly not be favorable. The most conservative estimate of time to stunning and death for medium-sized Baltic prawns heated from 10 °C were t_10->26 °C, core_ = 14.0 ± 1.2 s and t_10->44 °C, core_ = 24.25 ± 1.3 s. These times may appear intuitively longer than what would be expected, but they should also be considered in comparison with the less conservative estimates of stunning and “brain” death measured at the anterior ganglion t_26 °C, ant._ = 8.8 ± 0.5 s and t_44 °C, core._ = 18.0 ± 0.8 s. Approaching the exact heating times when a Baltic prawn is stunned and killed would require extensive experimentation with live cooking for short durations of time followed by cooling and attempted recovery, which lies outside the scope of this study on carcasses. However, it may be fair to assume that the true time to stunning and death may lie somewhere between 9–14 s and 18–24 s, respectively, for Baltic prawns of normal eating size with a realistic cooking regime of 1:9 crustacean to boiling water ratio. When adjusted to a warm room temperature of ~24 °C (a realistic in-house summer temperature in Northern Europe) and applied directly into boiling saltwater at 100.5 °C without addition of water to adjust to a normal cooking ratio, similar sized Baltic prawns heated faster with t_24->26 °C, ant._ = 2.3 ± 1.2 s and t_24->44 °C, ant._ = 9.3 ± 2.3 s (Table S2). However, this cooking regime does not reflect actual cooking well, so it mostly serves to demonstrate the minimum time to reach stunning and killing at the anterior ganglion. It does, however, also point to the importance of the initial temperature. It was clear in our measurements that higher starting temperatures resulted in shorter times to reach stunning and death (Figure 4 and Figure 5 and Table S2). In his classic, but much debated, paper in 1961, Gunter suggested heating lobsters slowly rather than dumping them directly into boiling water to provide what appeared by direct observations on behavior a more humane death [8,9]. Fregin and Bickmeyer, 2016, came to the same conclusion based on electrophysical measurements on nerve activity [21]. Following this logic, it would be desirable to bring the decapod body temperature slowly up to an elevated level before cooking to shorten the time of suffering as much as possible rather than cooling it down in, e.g., an ice bath as this will prolong the transition phase from life to stunning and finally death. Also, using cooking stoves with a higher heating effect can potentially decrease this transition time. These considerations on quick and efficient killing methods should be balanced with any potential effects of different treatments on meat quality, e.g., texture and flavor.

Whether the reported times to reach proposed stunning and killing temperatures by boiling in different decapods fall within the acceptable time range of dispatching an animal as quickly and efficiently as possible is a question for legislators. However, it may be useful to put these values into perspective. A potential alternative to live boiling small decapods like Baltic prawns could be electrocution, although to our knowledge there are currently no available small-scale systems for such small decapods. Tests of current commercially available electrocution systems designed for single medium-sized decapods are not fully conclusive in effective stunning and killing time. In three project reports (non-peer reviewed) an electrocution device operating at 110 V and 2–5 A was reported to effectively terminate all nerve activity in European lobster, Norway lobster *Nephrops norvegicus* (Linnaeus, 1758), European green crab and brown crab within 10 s [18,19,20]. Another study on European crayfish *Astacus astacus* (Linnaeus, 1758), Galician crayfish *Pontastacus leptodactylus* (Eschscholtz, 1823), European lobster and American lobster *Homarus americanus* H. Milne-Edwards, 1837, using the same device and electrocution parameters reported that electrocution induced an epileptiform seizure like state of increased neural activity that paralyzed the animals with a reversible absence of responsiveness to mechanical stimulation for 10–60 min [21]. Whether 10 s of electrocution should be considered an effective stunning or killing time for medium- to large-sized decapod crustaceans is thus not entirely clear. It is worth noting that to achieve similar stunning/killing times in much smaller decapods may require considerably more power, similarly to electrofishing working most efficiently on large individuals due to the larger voltage gradient across the length of the body and larger surface area [29,30,31]. The safety element of high-powered electrocution devices must thus be taken into consideration when comparing efficiency between live boiling and electrocution in, e.g., a household setting. Also, the high cost of a single-use device to electrocute decapods relative to the multi-use pot-and-stove required for boiling may also constitute a non-negligible limitation for consumer interest.

Another useful comparison of time to death in the small-scale recreational setting of boiling small decapod crustaceans like prawns is the comparison to other recreational activities involving the killing of wild animals. Recreational hunting of mammals and birds is widely accepted, although the process of killing in a hunting setting, usually by puncturing vital organs, such as heart, lung and/or liver, by a projectile leading to hemorrhage, hypovolemic shock and ultimately death, is slower than electrocution or bolt pistoling at a slaughterhouse. It is difficult to measure time to unconsciousness and death in realistic hunting situations, but a large game animal such as red deer *Cervus elaphus* Linnaeus, 1758, shot as preferred in the heart/lung region will often cover 50 m of ground and in some cases up to 150 m before falling over incapacitated and eventually dying [32]. With an estimated escape speed of 5–10 m/s, this yields time to unconsciousness of 10–30 s under ideal conditions, whereas less optimal shot placement can lead to considerably longer killing times (Kanstrup, personal communication). In larger species of deer and antelope, the modeled time to incapacitation, even with ideal shot placement, can approach 60 s [33]. Killing by shooting of a mammal cannot be directly compared with killing by live boiling of a decapod crustacean. It is, for example, difficult to assess whether fatal boiling is more painful than a fatal shot. Still, this comparison of time to unconsciousness potentially holds some value in that it provides information about a generally acceptable time of induced pain, distress and suffering during recreational killing of beings that are generally considered sentient such as mammals [34]. In this regard, all sizes of Baltic prawns and even signal crayfish up to medium size (31.7 ± 6.4 g) included in this study and heated from ≥10 °C fall below 60 s of heating time until reaching the proposed stunning (incapacitation) temperature of 26 °C in the core.

A clear limitation of the present study is the use of euthanized decapod crustaceans rather than live individuals, in that actual stunning and killing temperatures were not easily observable and could not be measured electrophysiologically [21,24]. This decision was based on the reasoning that we considered it unnecessary to expose live animals to thermometer implantation and boiling when heating profiles can also be obtained from carcasses of euthanized animals. The selection of a stunning temperature of 26 °C and killing temperature of 44 °C was based on previous studies in which only the brown crab was also included in the present study [14]. However, should more precise stunning and killing temperatures arise for the included species via behavioral (e.g., lethal temperature 50% (LT50) experiments) or electrophysiology studies, then time to stunning and killing can easily be recalibrated by visiting the heating tables (Table S2). An interesting comparison between time to stunning can be made by revisiting a study by Fregin and Bickmeyer, 2016. These researchers found a decline in electrophysiological signals (i.e., anesthesia/stunning) at the abdominal ganglion of lobsters after 46.9 ± 11.6 s for juvenile lobster (23.1 ± 8.2 g) and 154.7 ± 30.3 s for adult lobster (635 ± 78 g) following transfer of the animals from 7 °C water to boiling water [21]. Although this temperature was a little lower than the 10 °C used in the present study, body sizes were comparable to the medium-sized signal crayfish (31.68 ± 6.35 g) and medium-sized lobsters (569.70 ± 183.66 g) used in our study, in which the time to stunning was quite comparable at t_10->26 °C, core._ = 47.8 ± 16.6 s and 181.0 ± 25.6 s, respectively. This indicates that a stunning temperature of 26 °C in the two astacidean species may be an appropriate assumption.

In addition to measuring stunning and killing times, other temperatures of interest, e.g., in regards of cooking times and food safety concerns are also extractable from the heating tables of core temperature (Table S2).

## 5. Conclusions

Decapod crustaceans represent a highly variable order in terms of body size and shape. As body mass increases so does surface area, but the transfer of heat from the exterior and into the animal remains a slow process in large decapods. While the killing by boiling of large species such as lobster and brown crab may be considered inhumanely slow, for smaller species such as prawns, incapacitation and killing by boiling most likely happens within a similar time frame as using electrocution and within a shorter time span than in the recreational killing of large wild mammals during hunting. Given the practical limitations of alternative killing methods for small decapods at the household level and other small-scale settings, prudence is advised in the constructing of legislation on the killing of decapod crustaceans that takes into account the large variability of sizes and shapes within this order.

## Figures and Tables

**Table 1:** National legislation on dispatching decapod crustaceans via live boiling.

Country	Legislation on Live Decapod Boiling	Specific Legislation on Large Decapods	Specific Legislation on Small Decapods
**European Union**			
Belgium	(√)	(√) ^1^	(√) ^1^
Czech Republic	X	X	X
Denmark	(√)	(√) ^2^	X
Estonia	X	X	X
France	X	X	X
Germany	X	X	X
Hungary	X	X	X
Italy	(√)	(√) ^3^	X
Latvia	X	X	X
Lithuania	X	X	X
Poland	X	X	X
Slovak Republic	X	X	X
Slovenia	X	X	X
Spain	X	X	X
Sweden	X	X	X
**Europe—non EU**			
Norway	√	X	X
Swiss Confederation	√	√	√
United Kingdom	√	X	X
**Remaining world**			
New Zealand	√	X	X
USA	X	X	X

√ = legislation in existence for specific country, (√) = local non-national legislation in existence or comments to law, X = no legislation in existence. ^1^ Reference to the Swiss exclusion of Dendrobranchiata, Stenopodidea and Caridea in Advice of the Brussels Animal Welfare Council (24 June 2021). ^2^ Only lobsters mentioned in commentary to law by The Ministry of Food, Agriculture and Fisheries of Denmark (25 January 2022). ^3^ Lobsters mentioned in opinion by Istituto Zooprofilattico della Lombardia (29 July 2007).

**Table 2:** Biometric parameters for specimens included in heating experiment.

Species (Size Group)	n(n_sub_)	BM (g)	TL (cm)	CL (cm)	CW (cm)
Baltic prawn (S)	4	0.77 ± 0.14	4.45 ± 0.21	0.95 ± 0.06	N/A
Baltic prawn (M)	16(4)	2.37 ± 0.29	6.18 ± 0.26	1.40 ± 0.07	N/A
Baltic prawn (M), n.s.	6	2.41 ± 0.26	6.12 ± 0.34	1.42 ± 0.08	N/A
Signal crayfish (S)	4	3.09 ± 1.75	4.78 ± 0.97	1.70 ± 0.42	N/A
Signal crayfish (M)	12(4)	30.75 ± 4.43	9.94 ± 0.19	3.76 ± 0.18	N/A
Signal crayfish (L)	4	86.18 ± 2.83	13.25 ± 0.24	5.20 ± 0.08	N/A
Lobster (M)	3	569.70 ± 183.66	26.40 ± 3.38	9.60 ± 1.13	N/A
Green crab (S)	4	5.11 ± 1.26	N/A	N/A	2.65 ± 0.30
Green crab (M)	12(4)	49.46 ± 4.79	N/A	N/A	5.84 ± 0.17
Green crab (L)	4	70.55 ± 7.14	N/A	N/A	6.60 ± 0.42
Brown crab (S)	4	70.04 ± 8.36	N/A	N/A	7.58 ± 0.21
Brown crab (M)	2	704.35 ± 7.00	N/A	N/A	17.1 ± 1.27

BM = body mass, TL = total length, CL = carapace length (eye socket to posterior carapace), CW = carapace width, S = “small”, M = “medium”, L = “large”, N/A = not applicable, n(n_sub_) = number of individuals within each size group and number of individuals within any subgroups (0 °C, 10 °C, 20 °C, core), n.s. = non standardized heating. All values are average ± standard deviation.

## Data Availability

All data related to this study are available in figures, tables and Supplementary Materials. Imaging data of the diffusible iodine-based contrast-enhanced micro-CT imaged Baltic prawn and green crab are available at Aarhus University’s big data solution ERDA: https://anon.erda.au.dk/share_redirect/C9ExEJAmQz (Baltic prawn, 18.9 GB) and https://anon.erda.au.dk/share_redirect/F006tvJSSM (Green crab, 26.3 GB).

## Supplementary Materials

The following supporting information can be downloaded at: https://www.mdpi.com/article/10.3390/ani14223277/s1, Table S1. Geometric parameters for decapods included in the analysis of the correlation between body size and shape. Table S2. Biometric parameters and heating profiles for all decapods included in the heating experiments.
